
JOURNAL INFORMATION
==============================
NLM Title Abbreviation: Med Chir Rev
NLM Journal ID: 0034617

The Medico-Chirurgical Review

Publisher: Biomedical Journal Digitization Project

ARTICLE INFORMATION
==============================
PMCID: PMC4966254
PMID: 29917625
Article version: 1
Subjects: Reviews, Dublin Hospital Reports

Cases of Disease of the Heart, Accompanied with Pathological Observations

Print publication date: 1828 Oct 1
Volume: 9
Issue: 18
First page: 385
Last page: 399
1828 Sep
License: This work is free of known copyright restrictions. For more information, please see PMC Back Issue Digitization.
License URL: https://creativecommons.org/publicdomain/mark/1.0/

==============================

### XI.

*Cases of Disease of the Heart, accompanied with Pathological Observations.* By ROBERT ADAMS, A.B. one of the Surgeons to Jervis Street Infirmary. Octavo, pp. 102.

[Dublin Hospital Reports.]

IN this very extended paper, Mr. Adams has reported many remarkable examples of disease of the heart and its coverings, which have occurred in his own practice; and to these cases he has appended such observations as naturally arose in his mind, during a melancholy attendance on the sufferers. The facts and observations are very well arranged under three heads—Morbid Affections of the Membranous Coverings of the Heart—Changes in its Muscular Structure—Organic Alterations of the Auricular and Arterial Openings.

### I.—MEMBRANOUS COVERINGS.

The pericardium consists of two layers, widely different in structure and use. The internal, or serous layer, is a transparent membrane, forming a shut sac, investing the heart, and reflected thence over the internal surface of the external or fibrous portion. This last is partially covered by the pleura—attached to the diaphragm below—and lost above by surrounding the arteries. The fibrous portion of pericardium is, therefore, interposed between two very delicately organized membranes—and may probably serve as a barrier to *retard* the extension of inflammation from one serous membrane to the other—and thus prevent a combination of pleuritis and pericarditis. This combination, however, does not very uncommonly take place—nor is it easy to say in which membrane the phlogosis commenced. The pleura, from its position, is probably the first seat of inflammation in these cases.

A. *Fibrous Membrane.* This membrane is liable to ossification, which, however, is a rare disease. The only minute and full account of such a case is given by Laennec. The patient died dropsical, with the usual symptoms of diseased heart.

"On dissection the heart was found enlarged, and at first sight seemed as it were enclosed in a bony case; around the base of the ventricles there was a band about two inches broad, partly cartilaginous, partly bony, unequally thick, flattened, and a little rough on its surface, which sent from its interior a process, separating the ventricles from the auricles, and along each side of the septum of the ventricles, it produced a triangular prolongation, almost entirely cartilaginous, about two inches broad above, which terminated in an angle at a short distance from the apex of the heart; this plate of bone was evidently developed between the fibrous and serous layer of the pericardium." 356.

Mr. Adams met with one instance of this rare affection in Sir P. Dun's Hospital. The man had been suffocated by charcoal, and, on dissection, the heart was found adherent to the pericardium, and was surrounded by a zone of bone, three lines in thickness, and more than an inch in breadth.

B. *Serous Membrane.* The *anatomical characters* of pericarditis are well described by recent pathologists; but the symptoms, during life, are very equivocal and obscure.

"I have been frequently called upon to examine the bodies of persons supposed to have died of disease of the lungs or liver, and I have found pericarditis the true and single source of death, although during the previous illness the heart was never suspected to be the seat of a fatal affection. I have myself been witness to the treatment of a case of chronic inflammation of the pericardium in a girl aged 14, which terminated fatally, and during the whole progress of the disease phthisis, or rather what was considered hepatic phthisis, was the disease under which this child was supposed to be gradually declining. Her general appearance bore the character of scrophula; a few days previous to her death I took the following notes of her state, which I copy verbatim. 'Sarah Langley is greatly emaciated and very weak, yet does not wish to remain in bed, but prefers to be dressed, and to sit most of the day crouched by the side of the fire; the countenance is pale, and bespeaks great distress in the chest, yet the lips are not livid, but of a good color; the eyes appear large and brilliant; the skin is harsh, dry and rough, and cannot be by any means made clean; there is a general disposition to a growth of downy hair every where on the surface; the skin is never relaxed by perspiration; the bowels are regular, but occasionally affected by diarrhœa. The tongue is reddish, and but little different from the healthy state; the respiration is 54, the pulse 156 in the minute. There is much wheezing in her respiration during the night, and frequent cough, with but little expectoration. Upon exposing the chest and abdomen with a view to make a more particular examination, the former presented an unusually good formation; it was equally and uniformly convex, and the outlines of what we supposed a much enlarged liver could be traced through the parietes of the abdomen. The child lived about a week after these notes were taken.'" 361.

*Dissection.* The lungs were sound; the pericardium contained 20 ounces of purulent fluid. An organized membrane invested the heart's surface, and lined the reflected layer. It was granulated. The liver was sound, but depressed, in an unusual manner, into the abdomen, probably from the distention of the pericardium. The above was a case of chronic pericarditis, and the symptoms very puzzling. Let us look at a case of the acute kind.

A young lady had enjoyed good health till within a week of her illness, when she plunged her feet into cold water during the catamenial period. A slight pulmonic attack (as was supposed) succeeded, from which she apparently recovered. But her spirits drooped from this time, and she was averse to exercise. The next period passed without the menstrual secretion, and now she became oppressed in her breathing, with short cough, hot skin, pale countenance, small quick and irregular pulse, total loss of appetite—and, in short, many of the symptoms of ordinary fever, which compelled her to take to her bed. She lay on her left side or back, teazed with dry cough, and occasional paroxysms of dyspnœa, and some tendency to syncope. She seldom got more than two hours sleep at night. The treatment consisted of venesection, leeches, blisters, and purgatives. Anodynes were given to procure sleep. She died at the expiration of 14 days—the brain and lungs were the supposed seats of the disease.

*Dissection.* "Upon raising up the sternum a large mass of yellow tenacious lymph covered the pericardium, and filled the cellular membrane which connects this bag to the back part of the sternum. Upon cutting into the pericardium a quantity of sero-purulent fluid flowed out, the whole of the heart's surface was covered with a thick tenacious layer of organized lymph, rough, and reticulated on its surface. The concavity of the pericardium presented the same appearance, and several minute red specks denoted the organized state of the new membrane, which had been produced by very active inflammation, yet the exact seat of which had never once been suspected by two very eminent medical gentlemen who had seen this lady in her short illness. The lungs in this case were perfectly sound; the vessels of the bronchial membrane, particularly at the bifurcation of the trachea, were in a slight state of congestion." 363.

The next case related is one of rheumatic metastasis.

A female child, 6 years of age, had a severe attack of acute rheumatism in several joints as well as muscles. Active depletion was employed, as well as the warm bath, and the disease was subdued. In a few months afterwards she was again attacked, and the constitutional symptoms were very severe. The remedies were active, but the symptoms changed little till the fourth day—

"About which time the inflammation leaving its original seat passed along the muscles forming the parietes of the abdomen and thorax, and in the latter region very particularly fixed itself on the left side over the heart." 366.

The pulse now presented a remarkable tremulous vibratory feel, in addition to hardness and frequency. The horizontal posture could not be borne—the pain had left the limbs—there was great distress about the heart—breathing hurried—countenance anxious—great debility—some cough—viscid expectoration. She died on the 18th day of her illness.

"*Dissection.* The body was remarkably pale, and had rather a bloated appearance. The abdomen, particularly examined, exhibited no trace of inflammation. The lungs were perfectly healthy; but the pericardium was much enlarged and evidently distended by a fluid. On opening this bag, a quantity of sero-purulent fluid, with flakes of lymph floating in it, poured out. The surface of the heart and corresponding part of the pericardium were coated with lymph, and presented the usual appearances of acute pericarditis." 367.

Mr. A. draws the attention of his brethren to the distinction between simple rheumatism of the heart, and rheumatic inflammation of that organ. The following is our author's description of the former affection.

"In the one case the organ is simply, and often but transiently affected, just as any other muscle is, the person has perhaps been affected with rheumatic pains in the loins, with but little fever; these suddenly leaving this region run to the diaphragm, and cause a temporary affection of the breathing, with what the patient calls spasms in the chest. The countenance undergoes sudden changes: there are at such moments strong beats of the heart, and intermissions of the pulse sensible to the patient; and in females I have sometimes seen such attacks end in an hysteric paroxysm, and all symptoms subside when the lumbar pains returned. In such cases, the tongue is somewhat foul, the skin is frequently relaxed by profuse perspiration, and the urine is remarkably turbid; but the pulse has neither the frequency, hardness, nor peculiar vibratory feel that it has in the other, and more dangerous case: the countenance does not betray that anxiety, or, as it is denominated by some authors, that anguish which it almost uniformly expresses when the membranes of the heart are affected with acute inflammation, from whatsoever cause proceeding." 369.

Although this may occasionally pass on to carditis, there cannot, our author thinks, be a doubt that the two cases are very different, and require different treatment. Carditis rheumatica Mr. A. has usually seen in children, and in persons about or under the age of puberty, in whom metastasis is more liable to occur than in advanced life. "The translation of rheumatic inflammation to the heart has usually occurred where the synovial system of the extremities was the original seat of the disease."

Our author is doubtful whether there has ever been any fatal case of metastasis, of true arthritic inflammation, to the serous membrane of the heart, though this disease undoubtedly affects the heart very often, exciting agonising pain in the breast. He has known two cases of erysipelas of the head and face terminate fatally by metastasis to the heart.

"In both, as soon as the external redness receded, the breathing became distressed, the countenance agitated, and rest in the horizontal position impossible. Yet the general symptoms of pericarditis were so obscure, as to excite no suspicion in the mind of the attendants as to the real nature of the case. Both were weak and debilitated patients, in which this affection supervened towards the close of a long illness: neither lived more than two days after the sudden disappearance from the surface of the erysipelatous redness. The countenance, the breathing, the state of the pulse, and apparent debility in both, seemed to forbid any active interference on the part of the medical attendants, who had no suspicion of the real nature of the case, (so obscure were the symptoms of pericarditis) until the examination of the body disclosed it. The pericardium contained some turbid fluid, but had contracted no adhesions to the heart. A quantity of lymph, evidently recently effused, lined the concavity of the pericardium, and thickly and unequally covered the surface of the heart itself: appearances which left no doubt as to the immediate cause of the death of these individuals." 372.

### II. Changes in the Muscular Structure.

The parietes of the heart may become increased in thickness, and its cavities enlarged, with or without any ostensible cause. The usual ostensible cause is a narrowing of the aperture through which the blood passes out from an auricle or a ventricle. Sometimes, however, the organ attains a great size without this cause. The following is a remarkable instance.

A medical gentleman, æt. 60, of very anxious mind, had enjoyed good health, *with the exception of three rheumatic fevers*, from the last of which his chest suffered severely, and was left very susceptible of coughs and colds. He had palpitation—difficulty of breathing—bad digestion, though the appetite was good—flatulence—disturbed uneasy sleep—inability to lie on the *right* side—pulse 70, but very strong and full. The chest was well formed, but the action of the heart was inordinately strong, and extended over the whole left side of the chest. The difficulty of breathing was not constant—it came on in violent paroxysms, which were relieved by leaning forward. All these symptoms increasing, his legs began to swell—the abdomen filled with water—the pulse intermitted—he became comatose, and died.

*Dissection.* The left lung was compressed and condensed in an extraordinary degree—no water in the chest—the heart was double its natural size in every respect—its cavities enlarged in proportion to the thickening of the parietes—but no other disease of any kind. The dissection was made by Mr. Wilmott. The patient derived great benefit from occasional venesection.

Another case, the symptoms of which are almost exactly similar, but much more detailed, is given by our author. In this case there had been no preceding rheumatism, but the patient had one severe and profuse epistaxis—and afterwards a violent pneumonia, requiring the loss of 60 ounces of blood in one day. The phenomena of enlargement of the heart were equally as prominent as in the preceding case; but still his physicians had hopes that many of his symptoms were nervous. After various vicissitudes, the patient was seized with fever, which reduced him to the door of death; yet, strange to say, he not only weathered this storm, but nearly lost his cough, dyspnœa, and other symptoms of pulmonary and cardiac disease. The mitigation of his disease was only temporary, and mainly attributable to the depletion and abstinence employed during the fever. Still, from time to time, he was greatly relieved by venesection, diuretics, and low diet.

"In this way was this gentleman's life prolonged, every symptom was palliated as it presented itself, the cough and dyspnœa were always more or less relieved by venesection: the swelling of the limbs would remain obstinate for a time, and general dropsy be apprehended, then the kidneys would act, and the quantity of urine which would be evacuated was surprising. We ascertained that for several nights in succession above four quarts of urine were evacuated: at this time the thirst was not urgent, and he drank but little; the œdema of the feet and legs, after these extraordinary evacuations, would totally disappear, and perhaps for weeks there would be no return of the swelling." 383.

So long as the gentleman strictly adhered to vegetable regimen, the cough, dyspnœa, and palpitation were not troublesome; "but the slightest deviation from the general plan laid down did not fail to be followed by some threatening of his former symptoms." In one of his relapses, venesection failed to give even a temporary relief—diuretics made no impression on the kidneys—the abdomen became distended—symptoms of hydrothorax came on—and an unexpected attack of peritonitis put a period to his life.

*Dissection.* The peritoneal surfaces of the abdominal organs presented the usual appearances of inflammation. The structure of the liver was not diseased.

There were hydatids in one of the kidneys. The right cavity of the thorax contained about seven pints of whey-coloured serum. Two thirds of this lung were converted into a solid substance that sunk in water. The other lung was sound. The heart and pericardium almost universally adhered.

"The heart itself was fully three times its natural size: the parietes of both ventricles were greatly thickened, but particularly the left; at one spot only, towards the root of the pulmonary artery, the right ventricle appeared thin and weak. The muscular structure of the interior of the organ was much developed. The carneæ columnæ of the right side were remarkably prominent, and those of the left also were greatly thickened and enlarged. The valvular apparatus of both sides was perfect: we could discover no bony or earthy deposition either in the heart or in any part of the arterial system. All present agreed that they had never seen a heart so much enlarged." 387.

Mr. Adams believes that active hypertrophy of the heart is occasionally the cause of apoplexy—and, of this, we think, there cannot be a rational doubt entertained. Mr. A. observes, that the attention of the physician has not been sufficiently directed to the fact "that apoplexy may be the result of a state of the heart, altogether different from that we have been just considering." This is a change of the muscular structure into fat. There can be no question that the brain may become oppressed in two very different ways—by arterial turgescence, and venous remora, congestion, or stasis. The following interesting case we shall present in the words of the author.

"An officer in the revenue, aged 68 years, of a full habit of body, had for a long time been incapable of any exertion, as he was subject to oppression of his breathing, and continued cough. In May, 1819, in conjunction with his ordinary medical attendant, Mr. Duggan, I saw this gentleman: he was just then recovering from the effects of an apoplectic attack, which had suddenly seized him three days before. He was well enough to be about his house, and even to go out. But he was oppressed by stupor, having a constant disposition to sleep, and still a very troublesome cough. What most attracted my attention was, the irregularity of his breathing, and remarkable slowness of the pulse, which generally ranged at the rate of 30 in a minute. Mr. Duggan informed me that he had been in almost continual attendance on this gentleman for the last seven years; and that during that period he had seen him, he is quite certain, in no less than twenty apoplectic attacks. Before each of them he was observed, for a day or two, heavy and lethargic, with loss of memory. He would then fall down in a state of complete insensibility, and was on several occasions hurt by the fall. When they attacked him, his pulse would become even slower than usual; his breathing loudly stertorous. He was bled without loss of time, and the most active purgative medicines were exhibited. As a preventive measure, a large issue was inserted in the neck, and a spare regimen was directed for him. He recovered from these attacks without any paralysis. Œdema of the feet and ankles came on early in December; his cough became more urgent, and his breathing more oppressed; his faculties too became weaker.

"November 4th, 1819, he was suddenly seized with an apoplectic attack, which in two hours carried him off, before the arrival of his medical attendant." 397.

*Dissection.* There was a gelatinous fluid between the arachnoid membrane and pia mater. The substance of the brain was watery, and of a yellowish white colour. There was some water in the ventricles. "The coats of the carotid and middle artery of the dura mater were quite white and opaque from bony deposition, but were pervious."

"The right lung was sound. The left was compressed, and adhered to the side of the thorax: about a pint of serum and quantities of soft fat, of a very deep yellow colour, filled up the space between the anterior mediastinum and the compressed lung, which was impervious to air, and must have been totally useless.

"The right auricle of the heart was much dilated. The right ventricle externally presented no appearance whatever of muscular fibres: it seemed composed of fat through almost its whole substance, of the same deep yellow colour as that which occupied the place of the left lung. The reticulated lining of the ventricle, which here and there allowed the fat to appear between its fibres, alone presented any appearance of muscular structure.

"The left ventricle was very thin, and its whole surface was covered with a layer of fat. Beneath this, the muscular structure was not a line in thickness; it had degenerated from its natural state; was soft, and easily torn, and a section of it exhibited more the appearance of liver than of a heart. The septum of the ventricles presented the same appearance. In both ventricles, even in the lining fibres, yellow spots, where fat had occupied the place of muscular structure, were to be observed. The whole organ was remarkably light; the valves were all sound, except those of the aorta, which were studded with specks of bone, but elsewhere were cartilaginous and elastic, from which they derived a disposition to remain closed; a fluid gently injected from the ventricle would pass them; still, when the heart was reversed and water poured from the ventricle upon them, their valves retained it; its weight was not sufficient to separate the edges of the thickened valves. There was much fluid blood contained in the heart." 399.

The liver was natural—the spleen enlarged—the other viscera sound.

Passing over some cases of rupture of the ventricles, we come to a curious case communicated to Mr. Adams by Dr. Cheyne.

*Case.* A musician, aged 34 years, robust, sanguine, and intemperate, was seized with a most acute pain exactly in the region of the heart. At one time it was so violent as to render him nearly frantic, and five or six persons were required to hold him in bed. He had a dry cough—his breathing was oppressed. He recovered in a great degree from this; but after exposure to cold, and imprudent exercise, he relapsed, and presented himself to Dr. Cheyne with œdematous legs and thighs.

"The stroke of the heart was indistinct, tremulous, and appeared to extend over the whole of the left side of the chest, from above the clavicle to below the scrobiculus cordis; at no one part between these points was the stroke more distinct than at another. His pulse was 148, unequal, irregular, and indistinct; his complexion was of a leaden colour, his countenance bloated, his eye staring and wild. His recollection was becoming indistinct; unable to lie down, he passed the night in his chair. His appetite was not much impaired, but he was flatulent and costive; his tongue was furred, its edges were livid. His urine was scanty, high coloured and lateritious." 406.

He died three days afterwards without a struggle. There were several quarts of fluid in the right thoracic cavity—also a considerable quantity in the pericardium—lungs sound—the heart was as large as that of a bullock.

"The parietes of the left ventricle were thickened, its internal surface much inflamed, various irregular excrescences grew from the mitral valves and semilunar valves of the aorta, and the cordæ tendineæ, which connect the larger portion of the mitral valve to the walls of the left ventricle, were torn off just at the point of their insertion into the edge of the valve; at this point there were also some of the abovementioned excrescences; four of the broken cordæ tendineæ hung loose into the ventricle." 406.

One case is given by Mr. Adams of false aneurism of the heart, namely, a pouch going off from one of the ventricles. Most of the cases on record, of this rare disease, have already been presented to our readers in this Journal. The following will complete the list, up to the present time.

A female, aged 39 years, of sanguineous temperament, but with a constitution ruined by intemperance and profligacy, was admitted into one of the Dublin hospitals; but, being deaf and dumb, no accurate history of the complaint could be obtained. Her pulse was full and quick—tongue white—breathing difficult—lips livid—pointed to the region of the heart as the seat of her sufferings. She was bled, blistered, purged, and starved; by which means she was so much relieved that she left the hospital in ten days. Not long afterwards she returned, with all her former symptoms now greatly aggravated. The action of the heart was seen through her dress, and she could not bear the least pressure on that region—countenance bloated and livid—great difficulty of breathing—feet and limbs anasarcous—pulse feeble and intermitting. She died three or four days after admission.

*Dissection.* On raising the sternum, Mr. Harrison (demonstrator of anatomy) was struck with the unusual size of the pericardium, its opacity and great firmness, particularly towards the apex of the heart, where it was distended by a tumour of considerable magnitude. The left lung adhered to the pericardium, and the phrenic nerve was imbedded in a quantity of adhesive matter, (the result of inflammation) connecting the lung to the pericardium—and thus thrown a good deal out of its natural course. The pericardium was unusually adherent to the heart. There was a large round tumour, of very firm consistence, situated behind and below the apex of the heart. It was nearly as large as the heart itself, and much more firm to the touch.

"It was inseparably attached to the left ventricle, and to the pericardium; in some parts it was as firm as bone, and small patches of calcareous matter could be felt in different situations. Inferiorly the tumour rested on, and was closely attached to, the central tendon of the diaphragm; anteriorly it was intimately connected with the pleura, the cartilages of the 5th, 6th, and 7th ribs, and the intervening muscles; this latter connexion required to be cautiously dissected through, as the sac was here very thin, though as hard as bone. Upon making a small incision into the tumour posteriorly, I found that the sac was very thin, and similar to that of an old arterial aneurism; it was lined with a stratum of chalky or calcareous substance, which in some spots was so firm as to resist the knife; the sac in this situation was about the thickness of a wafer, and appeared to be formed solely of the condensed and altered pericardium; elsewhere the sac was found much thicker, and appeared to have been formed not only by the pericardium, but also by the fleshy substance, and lining membrane of the heart; in some places the fleshy fibres, after a short course, became so condensed and pale as to lose all appearance of muscle; in all other situations the pericardium and lining membrane of the heart were closely connected, except where, in a few small patches, some earthy depositions intervened; at the lowest part of the tumour the sac appeared to be formed solely of the thickened pericardium." 411.

From this examination, Mr. Harrison inferred that the aneurismal sac had been formed, in the first instance, by dilatation of the three structures; but that, as the tumour increased, the two inner structures became absorbed, so that the pericardial covering only remained. The sac was filled with a firm coagulated mass, of greyish-colour and laminated texture, as in a common aneurism. There was nothing unusual in the right or left auricle, or in the auriculo-ventricular valves. The right ventricle was sound. The lining membrane of the left ventricle was unusually dense and white—the muscular structure natural—at the apex was the aperture leading to the aneurismal sac, filled with a clot of blood. On making a section of the tumour, it was found that a considerable portion of its parietes was formed by a dilatation of the muscular substance of the heart. The coagulated mass occupying the tumour was formed of successive layers of coagulated blood. There were no morbid appearances in the abdomen.

### III. ORGANIC CHANGES IN THE ARTERIAL AND AURICULAR OPENINGS OF THE VENTRICLES.

These apertures are liable to contractions invariably combined with cartilaginous or osseous depositions. Such alterations much disturb the circulation, and through that the function of almost every organ in the body. The right side of the heart is infinitely less frequently affected in this way than the left. Our author has never seen the right auriculo-ventricular opening contracted; whereas the left aperture is, he thinks, much more often affected than is generally imagined. This disease occurs at all periods of life—in the young as well as in the old—as often in females as in males. The signs of this complaint, in its early stage, are so anomalous, that they are usually denominated nervous, and the treatment is too often calculated to aggravate rather than relieve the organic complaint. We have seldom an opportunity of investigating the early changes which take place in these apertures, as the patient rarely dies until worn out by a long train of suffering, and until the aperture of communication between the left chambers of the heart is converted into a mere fissure. Mr. A. had two opportunities of seeing this disease in its early stage, in consequence of sudden death, one by epilepsy, the other by apoplexy. We shall introduce some particulars of these cases.

*Case* 1. A lady died suddenly of apoplexy, as was said, at the age of 45. In the brain there was no effusion of blood or serum in the ventricles, nor any unnatural appearance, except a slight effusion between the membranes.

"The heart appeared remarkably short and was rounded towards its apex; in the interior of the organ every thing was natural except the mitral valve, which as yet, was not beset with earthy concretions, but was shortened to more than half its natural depth. It was yellow, opaque, and at the same time thickened as if a cartilaginous substance had been deposited between its laminæ; the aperture which the edges of this valve circumscribed was sufficiently open to allow the blood a free passage from the auricle into the ventricle; but it was manifestly incompetent to perform the full office of a valve, or prevent a regurgitation of blood into the auricle during the contractions of the ventricle." 417.

This lady had complained for nearly a year of numerous ill-defined sensations about the præcordial region, which were termed nervous, and were attended with oppressed breathing, palpitation, and small pulse. Thus, while she appeared in good health, her pulse was 120 in the minute, and extremely weak. This state of things appears to have excited no alarm in the minds of her medical attendants, though it produced an anticipation in the mind of the patient, which was unfortunately realized.

*Case* 2. Anne Conroy, aged 45, had enjoyed tolerable health till within a year of the date of application, at which time she complained of weight in her right side—palpitation—uneasiness about the heart—occasional cough with frothy expectoration—great variation in the tone of her voice—paroxysms of dyspnœa—a certain degree of livor of countenance. In other respects her health seemed good.

"The symptoms which most distressed her were palpitation of the heart and vertigo, with which last was generally associated the awful idea that she was about to fall down dead. Upon laying my hand over the precordial region, I discovered that the action of the heart, as to force and frequency, was indeed extraordinary, whilst the pulse, felt at the wrist, was a mere thready stream, unequal and irregular, beating at the rate of 150 in a minute. There was not as yet the slightest disposition to œdema. There was no deformity of the thorax observable." 419.

This want of correspondence between the action of the heart and the pulse—induced suspicion of organic disease, and blood was ordered from the arm—the diet directed to be vegetable—while digitalis and squills, in small doses, were prescribed. None of these directions were complied with, and in less than a month our author was called to the patient, who was in a fit of epilepsy. The universal convulsion did not relax for a moment till death closed the scene, a few hours afterwards.

In the brain there was great venous congestion, and slight opacity of the arachnoid—some serous effusion in the right side of the chest—right side of the heart of greater capacity than the left—pulmonary artery dilated—aorta contracted—left ventricle diminished in size—mitral valve not half its ordinary depth, its borders shrivelled and puckered up as if a thread were drawn round them, and containing some spiculæ of bone. "It was manifestly incompetent to do more than half guard the aperture of communication between the auricle and ventricle." This aperture itself was contracted. This state of valve allowed a reflux of blood into the auricle at each ventricular contraction. We shall conclude this division of the subject with the following descriptive sketch.

"When the disease is fully established, the signs of the contraction of the left auriculo-ventricular opening are by no means doubtful. The person affected with it has the general symptoms of diseased heart; there are paroxysms of dyspnœa and hæmoptysis; much uneasiness also is experienced in lying in any but one position; sometimes the patient can only lie with ease on the right side; he more commonly prefers the left, but the cause of these varieties I am unable to explain. The palpitations of the heart are irregular, widely extended; they are seen underneath the lower extremity of the sternum, and the heart beats with considerable force against the side of the chest. As the disease advances, all the above-mentioned symptoms are aggravated; the limbs become anasarcous, and the abdomen fills with water. Towards the latter period of the disease I have seen jaundice set in, and in so many cases that I cannot look upon it as an accidental circumstance, but rather as a symptom occasionally to be found attending the very last stage of this disease. Strong pulsations are seen in the jugular veins, and there appears a general thrill through the branches of the arterial system as in aneurisms; when the ear is attentively applied to the side of the thorax, a very complex kind of movement, hard to describe, is heard,—a hissing purring noise as it has been denominated, caused by the transmission of blood through a narrow orifice, is in most cases very evident. The more decided symptoms of this affection are to be found in the peculiar irregularity and want of correspondence in the pulse, as felt at the wrist, and examined simultaneously at the heart; the latter often beats so violently against the sides of the thorax as to shake the patient in his bed, while at the same time the arterial pulse is small, weak, and irregular; indeed such is the state of the pulse in the arteries, that the physician attending to this only, and overlooking the state of the heart, might readily suppose his patient in the last extremity, and dread to have recourse to those remedies from which alone any relief is to be expected.

"This irregularity, want of correspondence, and disproportion between the force of the beat of the heart and the pulse as felt at the wrist, are not the only circumstances worthy of our attention; for such a set of symptoms are common to the contraction of the auriculo-ventricular opening, and the narrowing of the aortic aperture; but the pathognomonic sign of the former disease will be found in this, that the heart will sometimes give two, three, or even four beats in succession, which are not perceptible to the hand examining the pulse in the arteries; the pulse at the wrist is very peculiar, and a knowledge of its character will assist us much in forming our diagnosis. I know not how to describe it otherwise than by saying, that it appears to the person examining it as if there were two pulses, one slow and deliberate for two or three beats, which is succeeded by three or four rapid and indistinct pulsations; the heart upon the whole generally pulsates ten or fifteen times more in a minute than the artery, and its stroke is often more distinct in one arm than another." 424.

The progress of this disease is generally very slow—and its severity can be greatly mitigated by medical and dietetical treatment—provided the patient will submit to confinement, quietude, and strict vegetable regimen. "Even after the dropsical symptoms have shewn themselves, the patient may live for a number of years." It is surprising, as our author remarks, to what a small fissure the left auriculo-ventricular aperture may be reduced before death supervenes. The dropsy occurring in this disease, is, our author thinks, more easily managed than that which occurs towards the close of any organic affection of the liver or lungs. We shall abridge one or two cases in illustration.

*Case.* Jane Gibson had great difficulty of breathing, constant palpitation, head-ache, vertigo, anasarcous limbs, abdomen ascitic, scanty urine, little or no cough; but the dyspnœa, &c. greatly aggravated by the least exertion. She could only lie on the right side. She had been about a year in this condition, having previously enjoyed good health. On examination, the chest was found well-formed—the action of the heart rapid, strong, and irregular—the pulse weak and thready—"*there were often two, three, or even four pulsations of the heart, at a moment when all pulsation was suspended in the arteries, and could not be felt by the finger.*" The pulse generally ranged to the number of 120 at the wrist—but more at the heart. The pulsation of the jugular veins was tremendous, and corresponded exactly with the action of the heart.

"Having seen such an exact combination of symptoms as this in many cases which terminated fatally, and ascertained by an examination after death that their source was to be found in the narrowing of the aperture of communication between the left auricle and ventricle, I have little doubt as to the real nature of this case." 427.

The woman is still living, but there can be little doubt as to the pathological condition of the heart. The want of correspondence between the ventricular action and the pulse at the wrist is an almost infallible sign of valvular disease, or disease of the apertures of communication between the chambers of the heart.

*Case.* A young lad, aged 15, had been delicate from infancy, and liable to dyspnœa, cough, palpitation, &c. from very slight causes. He had not been confined to the house, however, till within the last year. His urine became scanty—his limbs anasarcous—and he had, every evening, a paroxysm of dyspnœa.

"When I saw him, which was about six weeks before his death, I found him in the following state: his countenance shewed the distress and difficulty of breathing under which he laboured; his eyelids were swollen with a watery effusion; his inferior limbs were anasarcous: there was an unusual prominence of the left side of the sternum to be observed: the pulsations of the heart extended themselves widely over the thorax, and at a distance could be heard beating with considerable force against his breast. That this organ performed some kind of complex double movement, and that its motions were accompanied with a purring sound, as in varicose aneurisms, was plainly audible, and could be also recognized when the hand was placed over the region of the heart; the pulse at the wrist was very weak and irregular, sometimes very obscure, and always manifestly disproportioned to the powerful action of the heart. There was often a double pulse at the heart for the single beat in the arteries; pulsations were also seen in the veins of the neck. Upon a careful consideration of all the symptoms, I concluded that the contraction of the left auriculo-ventricular aperture, if not congenital in this case, had been for a long time completely established; the prognosis given was unfavourable; all that remained to be done was to palliate symptoms as they occurred." 429.

The dropsical swellings were often evacuated by diuretics, but all means at length failed to give relief, and death closed the scene.

*Dissection.* The lungs were sound—a pint of water in each pleural cavity—very little in the pericardium. The heart had an unnatural rounded appearance. The left ventricle was not near so long as the right, which terminated in a rounded pouch-like extremity, not at all resembling the natural apex of the heart. The two arteries were in proportion to the ventricles from which they arose, the pulmonary trunk being dilated to nearly twice its natural size; while the aorta was diminished to about one half its ordinary calibre. The cause of all this will now be seen.

"The left auricle was largely dilated, and contained a quantity of grumous blood; the left ventricle was of its natural thickness, but shortened and diminished a little in capacity. The communication between these was greatly interrupted, not by any contraction resulting from bony depositions, or by narrowing from any cause commencing in the zona annularis of the ventricle; but it appeared as if the contiguous edges of the left auriculo ventricular valves had, as it were, coalesced (or had never been separated); and thus was formed a transverse septum, constituting at once a floor to the auricle, and roof to the ventricle,—concave towards the former, convex towards the latter; perforated by an oblong opening, bearing in its appearance some resemblance to the rima of the larynx. There was not to be discovered in the valves a speck of bony or earthy deposition. A few yellow cartilaginous excrescences, preparatory to such a state, alone occupied the broadest extremity of the narrow opening." 432.

### IV.—DISEASE OF THE SEMILUNAR VALVES.

There is no more common organic lesion about the heart than ossification—at least, induration of the aortic valves. This change obstructs the free issue of blood from the ventricle, and prevents, of course, the valves from shutting when the ventricle is dilating. When this induration takes place gradually as old age creeps on, it is wonderful what little disturbance in the system at large is produced. But, on the other hand, when it occurs in young people (which is not very common) or about the middle period of life, the symptoms are well marked.

"There are strong palpitations of the heart, and dyspnœa complained of, which are much encreased by the slightest exertion; in a word, the ordinary signs of active enlargement of the left ventricle are present, except that the force of the arterial pulse is not proportioned to the action of the heart. It is irregular, and its contractions are accompanied with an hissing or purring sound, which can be clearly heard when the ear is applied to the side of the thorax, and a peculiar thrill is felt as in varicose aneurism, when the hand is laid on the præcordial region. With such a combination of symptoms this disease may be readily confounded with that which consists in a contraction of the left auriculo-ventricular opening, which we have just been considering: it is said, however, that the diagnosis will be found in this circumstance, that in the latter disease there is generally a double pulse to be felt at the heart for the single pulsation in the artery." 442.

When the heart is examined in those who die of this disease, viz. contraction of the arterial opening of the left ventricle, the parietes of this side are generally found hypertrophied, and the cavity increased in magnitude. We shall give some particulars of one more case before we close this article.

Case. A gentleman, aged 68, of pallid countenance, yet full and corpulent, while exerting himself in arranging some books, felt a sudden pain in his chest, extending down his right arm, accompanied by a sensation of numbness. His sight became dim—he had vertigo—but did not fall. From that time his breathing became oppressed—and the pulse in his right arm was imperceptible. It was extremely weak in the left radial. On the following day, 18th October, the most careful examination could not detect pulsation in any artery of the body. The movement of the heart was not perceptible by the hand on the chest. An obscure undulating motion was audible when the ear was applied to the cardiac region. His breathing was high and laborious, and could only be performed when the body was erect. At night he became worse—he was restless—and the attempt to lie down increased the dyspnœa. He was aware of his danger, being himself a physician, but he was tranquil, and even cheerful. His appetite was indifferent, and his digestion impaired. In this state he remained six weeks, with little alteration, except decline of strength and increase of dyspnœa. "During the entire of this distressing period, *no pulse was to be felt in any artery of the body*," although Mr. Adams made a daily and careful examination. At the end of the seventh week, after a restless and wretched night, he was observed to be a little delirious. He then fell into a state of stupor—"his upper lip became suddenly swollen, and a large livid spot appeared on it." At 12 o'clock that day he ceased to exist.

"*Dissection.* There was slight œdema of the lower limbs. The abdomen was much distended with air, and contained a small quantity of serum.

"There were adhesions of the right lung to the side of the thorax; on separating these, a cavity was discovered containing about a pint of a yellow sero-purulent fluid. This cavity was lined by a membrane of organized lymph, evidently of recent formation. The substance of this lung exhibited in no part any traces of inflammation. The left lung was not adherent; it was perfectly healthy; yet a small quantity of serum was contained in the left cavity.

"The heart was large, flabby, and of a yellow colour from fatty deposition: all its cavities were distended with fluid blood; the semilunar valves of the aorta were completely ossified; but this bony or earthy deposition was not confined to the aorta; it extended to the coronary arteries, which were so completely converted into bone as to be quite solid, having no perceptible cavity except at the distance of an inch from their origin: beyond this these vessels were at intervals completely interrupted by small bony specks." 447.

It will be acknowledged that the appearances above-mentioned do not satisfactorily account for the remarkable phenomenon of cessation of arterial pulsation for seven weeks. We have recorded a case which was under our own care, and where there was no pulsation in any tangible artery for 48 hours; but we never heard of one where the non-pulsation continued so long as in this case.* The hypothesis suggested by our author is as feasible as any one which we can imagine on this occasion, it is—"that the heart, directly deprived of its due

* Dr. Cheyne informs Mr. A. that he knows of two individuals, still living, in whom, for two days, there was a failure of the pulse. In one, which occurred during an attack of misplaced gout, the cessation was complete:—In the other, after an attack of cholera, a faint pulsation could be felt about four or five times in the minute.

supply of blood, by the obliteration of proper nutrient vessels, might have been suddenly thrown into a state of partial paralysis—hence the feebleness of its efforts, which were inadequate to excite the slightest movement in the arteries."

We have now endeavoured to exhibit a full and complete delineation of this paper, occupying more than one hundred pages of the volume before us. It does credit to the zeal and careful observation of the author, and must prove useful to his brethren at large. We return him our thanks for the information which he has afforded us.

---

### XII.
